# Microfluidic Technologies in Advancing Cancer Research

**DOI:** 10.3390/mi15121444

**Published:** 2024-11-28

**Authors:** Arjun Ajikumar, Kin Fong Lei

**Affiliations:** 1 Department of Biomedical Engineering, Chang Gung University, Taoyuan 33302, Taiwan; arjunajikumar852@gmail.com; 2 Department of Radiation Oncology, Chang Gung Memorial Hospital, Linkou, Taoyuan 33305, Taiwan; 3 Department of Electrical & Electronic Engineering, Yonsei University, Seoul 03722, Republic of Korea

**Keywords:** microfluidics, cancer research, droplet-based microfluidics, organ-on-chip, paper-based microfluidics, electrokinetics, immune response

## Abstract

This review explores the significant role of microfluidic technologies in advancing cancer research, focusing on the below key areas: droplet-based microfluidics, organ-on-chip systems, paper-based microfluidics, electrokinetic chips, and microfluidic chips for the study of immune response. Droplet-based microfluidics allows precise manipulation of cells and three-dimensional microtissues, enabling high-throughput experiments that reveal insights into cancer cell migration, invasion, and drug resistance. Organ-on-chip systems replicate human organs to assess drug efficacy and toxicity, particularly in the liver, heart, kidney, gut, lung, and brain. Paper-based microfluidics offers an alternative approach to accomplish rapid diagnostics and cell- and tissue-based bioassays. Electrokinetic microfluidic chips offer precise control over cell positioning and behavior, facilitating drug screening and cellular studies. Immune response studies leverage real-time observation of interactions between immune and cancer cells, supporting the development of immunotherapies. These microfluidic advances are paving the way for personalized cancer treatments while addressing challenges of scalability, cost, and clinical integration.

## 1. Introduction

Cancer is a leading global cause of mortality, characterized by uncontrolled cell growth and metastasis [1,2,3]. Its complexity arises from the diverse interactions between cancer cells and their microenvironment, often leading to immune evasion and treatment resistance. To address these challenges, microfluidic technologies have emerged as powerful tools in cancer research [4,5,6,7,8]. Microfluidics, the science of manipulating small fluid volumes within microchannels, offers a versatile platform for investigating cancer biology through a variety of specialized systems, including droplet-based microfluidics, organ-on-chip systems, paper-based microfluidics, electrokinetic microfluidic chips, and microfluidic chips for the study of immune response. These platforms provide precise control over cellular environments, enabling high-resolution, real-time observations and high-throughput analysis that closely mimic in vivo conditions. By replicating physiological scenarios, microfluidic technologies are transforming our understanding of cancer and paving the way for more effective treatments. This review highlights the crucial role of microfluidic technologies in advancing cancer research, with a focus on five main areas: droplet-based microfluidics, organ-on-chip systems, paper-based microfluidics, electrokinetic chips, and immune response analysis. A comparison of the microfluidic technologies in advancing cancer research is listed in Table 1.

Droplet-based microfluidics has revolutionized the study of cancer cell migration, invasion, and resistance by allowing individual cells or three-dimensional (3D) microtissues to be encapsulated within droplets. This enables researchers to conduct high-throughput experiments and observe cellular responses in real time, providing invaluable data for understanding cancer metastasis and testing potential anti-cancer therapies. Additionally, organ-on-chip systems have emerged as powerful tools for replicating the structure and function of human organs, offering physiologically accurate models to assess drug efficacy and toxicity. These models, which simulate organs such as the liver, heart, kidney, gut, lung, and brain, allow researchers to evaluate how different tissues respond to therapeutic interventions and identify potential toxic effects. Paper-based microfluidics is an emerging field in diagnostic and analytical sciences, where microfluidic channels and reservoirs are created on paper to manipulate small volumes of liquid. This approach was extended to develop a 3D tissue model for cancer research. Electrokinetic microfluidic chips offer precise control over cell positioning and interactions within the microfluidic environment, enabling researchers to manipulate cancer cells and study their responses to various stimuli. This technology has been instrumental in advancing drug screening and understanding cellular behaviors at a granular level. Immune response studies have benefited from the integration of microfluidic platforms, which allow for real-time observation of immune-cancer cell interactions. By enabling the co-culture of immune cells and cancer cells in a controlled environment, these platforms provide insights into how immune cells recognize and attack cancer cells and how tumors evade immune surveillance. This has opened new pathways for developing more effective immunotherapies aimed at enhancing the immune system’s ability to combat cancer.

As microfluidic technologies continue to evolve, they hold the potential to further transform cancer research by enabling personalized medicine approaches, where treatments are tailored to the specific characteristics of an individual’s tumor. However, challenges such as scalability, cost, and integration with clinical workflows must be addressed to fully realize the potential of these technologies. By overcoming these obstacles, microfluidics can continue to drive forward innovations in cancer research, leading to more effective treatments and improved patient outcomes.

## 2. Droplet-Based Microfluidics

Droplet microfluidics has revolutionized biomedical research by providing a versatile and efficient method for studying cells and molecules in isolated environments [9,10,11,12]. This technology involves generating droplets through microfluidic devices, typically using T-junctions, flow-focusing mechanisms, or co-flow setups to create uniform droplets of aqueous solutions suspended in oil [13,14]. These droplets function as discrete, independent reaction chambers, ideal for applications like single-cell analysis, drug screening, and the investigation of complex biological interactions within 3D environments.

**Figure 1:**
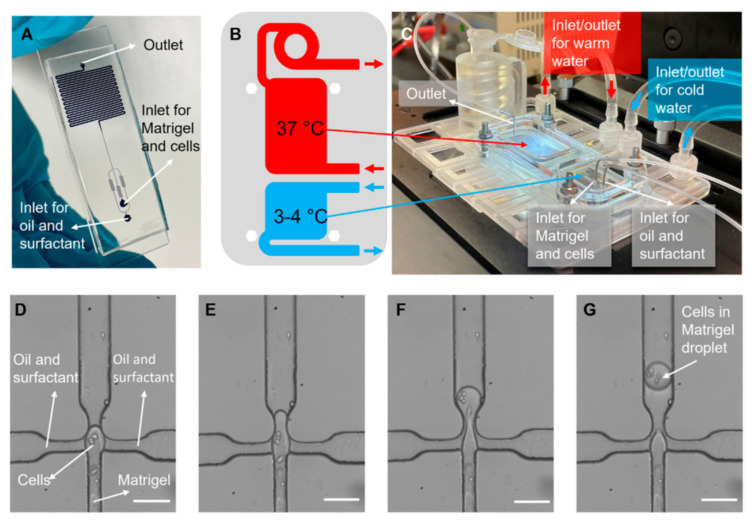
Cell encapsulation in the Matrigel process. (**A**) A picture of the microfluidic encapsulation chip, filled with blue dye. The flow-focusing section is visible at the bottom, and the meandering channel section for bead gelation is visible at the top. (**B**) A schematic view of two different temperature regions, and (**C**) a photo of the temperature control platform, showing how the chip is mounted onto the well-plate-sized platform. Warm and iced-cold connector lines are indicated with red and blue arrows, respectively. (**D**–**G**) Images of cell encapsulation in the flow-focusing junction, where Matrigel droplets encapsulating cancer cells are formed in oil. Scale bars: (**D**–**G**) 50 μm (Copyright © 2023 Jouybar et al. Reprinted from [15]).

The core advantage of droplet microfluidics lies in its ability to create highly controlled, miniature environments that mimic in vivo conditions. One of the key areas where this has been applied is in cancer research, particularly in studying the interactions between tumor cells and their surrounding extracellular matrix (ECM) [15]. By generating 3D microtissues inside droplets, researchers can closely observe how tumor cells behave in environments that resemble the body’s natural tissue structure. In Figure 1, cell encapsulation in the Matrigel process is shown in the droplet-based microfluidic device with two different temperature regions. The images of Matrigel droplets encapsulating cancer cells in a flow-focusing junction are also shown. In studies such as the development of 3D multicellular tumor spheroids, the technology has enabled a better understanding of how cancer cells grow, migrate, and resist drug treatment. Unlike traditional two-dimensional (2D) cultures, which fail to replicate the complexity of the tumor microenvironment, droplet-based 3D systems offer insights into the way ECM interactions influence cell behavior, particularly drug resistance. Moreover, another study reported encapsulating cancer cells in droplets within a 3D ECM, allowing them to observe how tumor cells invade and interact with different matrix compositions [16]. This approach revealed the significant role of the ECM in protecting tumor cells from chemotherapeutic agents, emphasizing the importance of considering cell–matrix interactions when developing cancer treatments. The use of droplet microfluidics to generate multicellular tumor spheroids, which demonstrated increased drug resistance compared to 2D cultures, was reported to further support the idea that 3D models offer more accurate representations of tumor behavior in response to therapies.

High-throughput drug screening is another key application of droplet microfluidics. For example, researchers encapsulated both cancer cell lines and human primary tumor samples within droplets to rapidly test multiple drugs in parallel [17]. The capability to perform such screenings in a miniaturized and isolated environment enables researchers to study how individual cells or cell clusters respond to treatments, leading to more efficient and scalable drug testing processes. This is particularly relevant for personalized medicine, where treatments must be tailored to the specific characteristics of a patient’s cancer. The high-throughput capability of droplet microfluidics makes it an ideal platform for testing a wide range of drugs on different cell types or tumor models in a relatively short amount of time.

In addition to drug screening, droplet microfluidics has proven highly effective in single-cell analysis. One study demonstrated the technology’s ability to isolate individual cells in droplets, allowing for a detailed examination of cell behavior at the molecular level [16]. This is particularly valuable for studying immune–cancer cell interactions, as it allows researchers to monitor real-time responses between immune cells and tumor cells at the single-cell level. Such insights are crucial for advancing immunotherapy, as they provide a clearer understanding of how immune cells can be engineered or stimulated to target and eliminate cancer cells more effectively. Moreover, droplet microfluidics has expanded the scope of mechanical stress analysis in cell migration studies. In one case, droplets were used as force sensors to measure the mechanical stresses exerted by migrating cells as they interacted with their environment. This quantitative measurement system provided valuable data on how cancer cells move and apply force during metastasis, contributing to a more detailed understanding of the physical dynamics involved in cancer spread [18]. By precisely measuring these forces, researchers can gain deeper insights into how mechanical factors drive cell migration, potentially leading to new therapeutic approaches that target the mechanical properties of tumors.

Despite its advantages, droplet microfluidics faces certain limitations. One of the primary challenges is replicating in vivo conditions over extended periods. While droplet-based models provide a highly controlled and isolated environment, they may fail to capture the complexity of interactions that occur within a living organism over time. Additionally, the scalability of these models remains an issue, as larger-scale applications or clinical settings require a balance between micro-level precision and macro-level applicability. Another challenge lies in modeling the tumor microenvironment with sufficient accuracy. While droplet microfluidics has enabled the creation of 3D microtumor models, as seen in one study assessing cell–ECM interactions and drug resistance, the complexity of these models still falls short of fully replicating human tissues [19]. The study found that the ECM played a significant role in drug resistance, with tumor cells in 3D environments showing higher resistance to chemotherapy than those in 2D cultures. However, the limited duration of experiments and the artificial nature of the ECM in these models suggest that more work is needed to make these systems clinically translatable.

Nevertheless, the programmable nature of droplet microfluidics offers exciting possibilities for future applications. The versatility, high-throughput potential, and precision of droplet microfluidics make it an indispensable technology in modern biomedical research. From enabling more accurate 3D tumor models to improving single-cell analysis and enhancing drug screening, the applications of this technology continue to expand. As researchers address the current challenges of scalability and long-term modeling, droplet microfluidics will likely play an even more critical role in advancing cancer research, personalized medicine, and therapeutic development.

## 3. Organ-on-a-Chip

Organ-on-a-chip technology represents a transformative approach in biomedical research, offering precise models that replicate the structural and functional intricacies of human organs [20,21,22]. These microfluidic devices recreate the microenvironment of tissues and organs, facilitating the study of physiological and pathological processes in a controlled and replicable manner [23,24]. By integrating living cells within these microenvironments, organ-on-a-chip systems enable real-time observation of cellular responses to various stimuli, making them invaluable tools for drug discovery, disease modeling, and personalized medicine.

**Figure 2:**
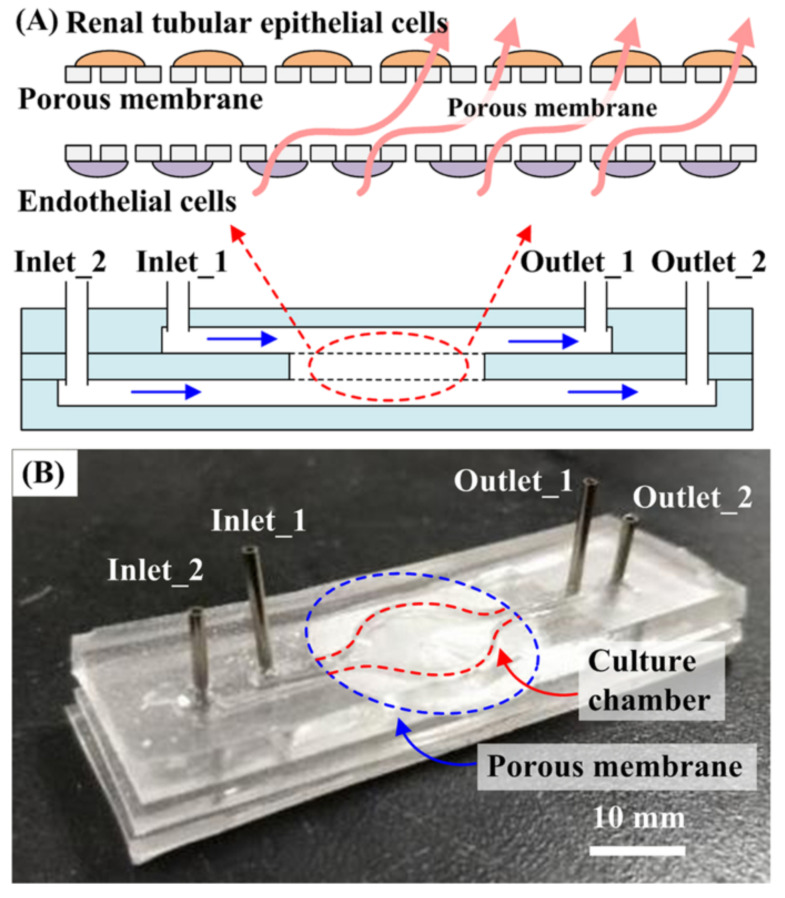
Design of the three-layer microfluidic kidney chip. (**A**) Schematic illustration of the chip. Renal proximal tubular epithelial cells (RPTECs) were cultured under physiological apical fluid shear stress, while peritubular capillary endothelial cells (PCECs) were cultured on the reverse side of the membrane. (**B**) The microfluidic kidney chip comprises two inlets, two outlets, two membranes, and three PDMS layers (Copyright © 2020 Yin et al. Reprinted from [29]).

One of the most impactful applications of organ-on-a-chip technology is in high-throughput screening. This method has revolutionized the simultaneous testing of multiple drug candidates by offering a more precise representation of human organ function than traditional 2D cell cultures and animal models. Liver-on-a-chip models are a prime example, replicating hepatic metabolism and detoxification processes, enabling researchers to assess the hepatotoxicity of drug candidates with greater reliability [25]. These models incorporate hepatocytes and other liver cell types within a microfluidic environment that mimics the liver’s natural architecture and functions, such as bile production, enzyme activity, and metabolic processes. This level of physiological relevance is crucial for studying liver diseases, including hepatitis, cirrhosis, and non-alcoholic fatty liver disease (NAFLD). By providing a more accurate simulation of the liver’s functions, liver-on-a-chip systems improve predictions of drug behavior in the human body, thereby enhancing the drug development process’s efficiency. Similarly, heart-on-a-chip technology has made significant advancements in replicating the beating function of the heart using engineered cardiac tissues [26,27]. These platforms incorporate cardiomyocytes, the muscle cells of the heart, into a microfluidic device that simulates the mechanical and electrical activity of cardiac tissue. Heart-on-a-chip systems are vital for investigating cardiac physiology, disease mechanisms, and the effects of pharmaceutical compounds on heart function. They provide significant advantages in studying conditions such as arrhythmias, myocardial infarction, and cardiomyopathies. Additionally, heart-on-a-chip models serve as powerful tools for evaluating the cardiotoxicity of new drugs, thereby enhancing the safety profile of potential therapeutics. The ability to observe real-time cardiac muscle contractions and electrical impulses within these chips allows for a detailed analysis of how drugs can affect heart rhythms and overall cardiac health. Moreover, kidney-on-a-chip devices have also emerged as crucial tools in biomedical research, replicating the filtration, reabsorption, and excretion functions of the kidney [28,29,30]. For example, the design of the three-layer microfluidic kidney chip is shown in Figure 2. The platform typically includes renal epithelial cells and other kidney-specific cell types arranged in a microfluidic setup that mimics the kidney’s nephron structure. Renal proximal tubular epithelial cells (RPTECs) were cultured under physiological apical fluid shear stress, while peritubular capillary endothelial cells (PCECs) were cultured on the reverse side of the membrane. Kidney-on-a-chip systems are invaluable for studying renal physiology, drug-induced nephrotoxicity, and kidney diseases such as acute kidney injury (AKI) and chronic kidney disease (CKD). By providing a more accurate model of human kidney function, these devices enable better predictions of drug effects and potential renal side effects, thus improving the overall drug development process. The nephron-mimicking structures in these chips allow researchers to study the precise mechanisms of kidney filtration and reabsorption, providing insights into how different compounds can affect renal function. Next, gut-on-a-chip technology has proven to be particularly valuable for studying the complex environment of the human intestine, including the epithelial barrier, mucus layer, and microbiome interactions [31,32]. These platforms use intestinal epithelial cells cultured in a microfluidic device that simulates the peristaltic movements and chemical gradients of the gut. Gut-on-a-chip systems are used to study nutrient absorption, drug metabolism, and interactions between the gut microbiota and the host. They also provide insights into gastrointestinal diseases such as inflammatory bowel disease (IBD), irritable bowel syndrome (IBS), and colorectal cancer. By offering a more realistic model of the human gut, these devices facilitate the development of targeted therapies and improve our understanding of gut health and disease. Researchers can observe how the gut microbiome interacts with intestinal cells and how various treatments can affect these interactions, leading to better strategies for managing and treating gastrointestinal disorders. Lung-on-a-chip models have been designed to replicate the structure and function of human lung tissue, including the alveolar–capillary interface, which is critical for gas exchange [33,34]. These platforms incorporate lung epithelial and endothelial cells in a microfluidic device that simulates breathing motions and airflow. Lung-on-a-chip systems are used to study respiratory diseases such as asthma, chronic obstructive pulmonary disease (COPD), and pulmonary infections. They are also valuable for assessing the pulmonary toxicity of inhaled substances and for developing new treatments for respiratory conditions. The ability to simulate the mechanical stretching and relaxation of lung tissues provides insights into how diseases affect lung function and how therapies can alleviate these effects. Brain-on-a-chip platforms aim to mimic the neural architecture and functionality of the human brain [35]. These devices integrate neurons and glial cells within a microfluidic environment that supports complex neural networks and synaptic interactions. Brain-on-a-chip models are used to study neurodegenerative diseases such as Alzheimer’s and Parkinson’s, as well as neurological disorders like epilepsy and traumatic brain injury [36,37]. These systems provide insights into neural development, neuroinflammation, and neurotoxicity, offering a powerful tool for drug screening and the development of neurotherapeutic strategies. The integration of neural cells within these chips allows for the study of electrical signaling and synaptic activity, providing a detailed understanding of brain function and dysfunction.

Despite the numerous advantages, organ-on-a-chip technology also faces several challenges. The complexity and cost of fabricating and operating these models are significant barriers to their wider adoption. The sophisticated engineering required to create and maintain these systems can be cost-prohibitive for many research institutions. Additionally, while organ-on-a-chip models are invaluable for detailed studies, scaling them up for high-throughput screening remains a challenge, potentially impeding their use in large-scale drug development processes. The intricate microfluidic designs and the need for precise control over environmental conditions can make these systems difficult to scale efficiently. Another critical issue is the integration of these models into standard pharmaceutical development pipelines. Ensuring compatibility with existing workflows and technologies is essential for broader applications, and this integration process is still ongoing. The development of standardized protocols and materials is necessary to reduce variability in the design and performance of organ-on-a-chip devices, which can affect the reproducibility and reliability of experimental results. Effective integration with downstream analytical techniques, such as imaging and omics technologies, is also crucial for fully exploiting the potential of these devices in biomedical research.

In summary, organ-on-a-chip technology has significantly advanced biomedical research by providing high-fidelity models that replicate human organ function. These platforms offer numerous advantages, including high-throughput analysis, physiological relevance, and versatility, while also addressing ethical concerns related to animal testing. Despite the challenges of complexity, cost, and standardization, ongoing advancements promise to enhance the robustness, integration, and applications of organ-on-a-chip systems. As this technology continues to evolve, it holds great potential for transforming drug discovery, disease modeling, and personalized medicine, ultimately improving human health and treatment outcomes. The future of healthcare looks brighter with the continued development and implementation of organ-on-a-chip technology, which stands as a testament to human ingenuity and the relentless pursuit of knowledge in the quest to improve human health.

## 4. Paper-Based Microfluidics

Paper-based microfluidics, known as microfluidic paper-based analytical devices (μPADs), is an emerging technology that leverages paper as a substrate for conducting various biomedical assays. This approach has gained considerable attention for point-of-care (POC) testing, particularly in resource-limited settings, due to its affordability, ease of use, and minimal equipment requirements. Numerous types of assays have been successfully demonstrated using μPADs, including colorimetric bio-assays [38,39,40], electrochemical bio-assays [41,42,43], and even paper-based enzyme-linked immunosorbent assays (ELISA) [44,45,46,47]. The advantages of μPADs, such as their lightweight nature, simplicity, and low cost, make them ideal for rapid screening applications at the POC applications. For instance, a paper-based ELISA assay can yield results within an hour, while traditional ELISA assays generally require at least six hours to complete [48]. Additionally, paper-based ELISA reduces sample and reagent volume requirements to as little as 3 µL per test zone, significantly lowering diagnostic costs compared to conventional methods.

**Figure 3:**
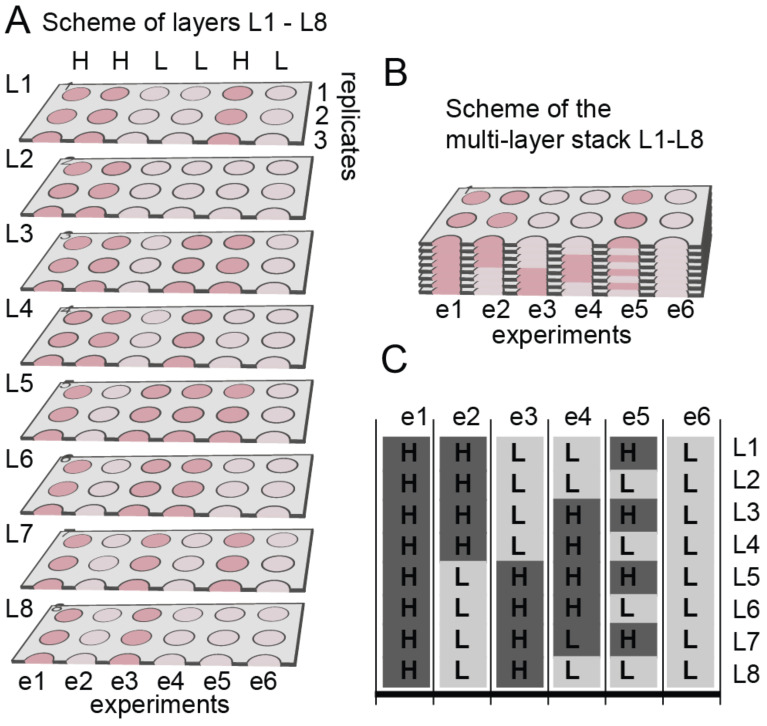
Scheme of the multi-zone, multi-layer sample containing high (H) and low (L) concentrations of cells in specific locations (red, H: 120,000 cells/zone or pink, L: 12,000 cells/zone). (**A**) Layers prior to stacking. Only three out of eight replicates are shown; to simplify visualizations. (**B**) Stacking the layers L1 through L8 generates L1L8-stack. (**C**) Distribution of GFP-MDA-MB-231 cells in layers L1 through L8. Dark grey color denotes zones that contain 120,000 cells/zone (“H”); light grey color denotes those that contain 12,000 cells/zone (“L”). Experiments are separated by vertical black lines (Copyright © 2011 Derda et al. Reprinted from [50]).

Building upon these capabilities, researchers have explored the use of paper-based microfluidics for more complex cell- and tissue-based bioassays [49,50,51,52,53,54]. The natural reticulated structure of paper enables the creation of a well-defined 3D space suitable for in vitro 3D cell cultures, where cells can be encapsulated in hydrogels and cultured within the paper substrate, as shown in Figure 3. Different cell concentrations were cultured in different locations of different layers. This multilayer paper-based approach allows the formation of oxygen and nutrient gradients, simulating tissue and organ-level functionality. Experimental observations have shown that this setup can create hypoxic conditions in the core of the paper stack, where cell populations exhibit growth arrest, apoptosis, or necrosis, closely mimicking the cellular microenvironments seen in tissues. To enhance the study of molecular expression, cells can also be cultured directly on filter paper without hydrogel encapsulation. This gel-free approach has been particularly useful for investigating signaling pathways and cellular phosphorylation. For example, immunoassays have been directly performed on filter paper following in situ cell fixation and membrane poration, with colorimetric results quantified using a commercial scanner [55,56,57]. In one notable study, the phosphorylation states of liver cancer cells were analyzed under different stimuli, such as IL-6 cytokine exposure, nutrient starvation, and hypoxic conditions, to assess kinase activation and study signaling pathway crosstalk. The simultaneous screening of over 40 kinases and transcription factors demonstrated the potential of this approach to explore complex cellular interactions with high specificity. The paper-based cell culture technique also facilitates the integration of multiple analytical processes on a single filter paper, reducing both the required cell and reagent volumes and the operation time. This approach has led to the development of sophisticated systems, such as a folding paper-based model designed to simulate the hypoxic microenvironment typical of solid tumors [58,59,60]. In this model, paper layers are arranged to mimic the inner and outer tumor regions, enabling differential analysis of protein expression across these layers. For example, hypoxia-inducible factor 1-alpha (HIF1-α) and vascular endothelial growth factor (VEGF) were highly expressed in the inner tumor layers, which promoted endothelial cell proliferation and migration, in contrast to the non-hypoxic outer layers.

In summary, the paper-based microfluidic platform presents a versatile, cost-effective tool for biomedical research. It offers an innovative approach to studying cellular behavior within a 3D microenvironment and tissue-mimicking structures, providing a favorable analytical method to investigate cell–microenvironment interactions with applications that span from disease diagnostics to cellular pathway analysis and tissue engineering. Looking to the future, several directions hold promise for advancing the application of paper-based microfluidics in cancer research. One key area is improving the detection sensitivity of these devices to rival traditional laboratory-based techniques. While paper-based platforms are cost-effective and portable, their sensitivity often falls short of established methods such as ELISA or PCR. To overcome this, researchers are exploring the integration of nanomaterials like gold nanoparticles, quantum dots, and plasmonic nanostructures into paper-based assays. These materials can amplify signals, enabling the detection of low-abundance cancer biomarkers, such as circulating tumor DNA, exosomes, or specific proteins, with high accuracy. Innovations in surface chemistry and fluid flow engineering can further enhance the reliability and reproducibility of these devices, making them viable alternatives to more expensive and resource-intensive lab tests. Another challenge lies in addressing variability in manufacturing and fluid dynamics on porous media. Paper, as a natural material, inherently varies in properties, such as porosity and surface texture, which can affect the performance of microfluidic devices. Advances in manufacturing processes, such as roll-to-roll printing and precision patterning using wax, inkjet, or laser methods, are paving the way for consistent and scalable production. Additionally, the development of hybrid substrates that combine paper with engineered polymers or coatings can provide more uniform fluidic behavior while maintaining the affordability and biodegradability of paper-based platforms. As paper-based microfluidics continues to evolve, the field has the potential to address major challenges in cancer diagnostics, treatment monitoring, and global health equity. Through a combination of scientific innovation, technological integration, and practical design, these devices can play a transformative role in advancing cancer research and improving patient care worldwide.

## 5. Electrokinetic Microfluidic Chips

Electrokinetic microfluidic chips represent an advanced and innovative approach in the field of cancer research [61,62]. These devices leverage electrokinetic phenomena, including electrophoresis, dielectrophoresis, and electroosmosis, to manipulate and control small volumes of fluids and particles at the microscale [63,64]. The precise control over the cellular microenvironment offered by electrokinetic microfluidic chips enables researchers to perform a wide range of experiments that can significantly enhance our understanding of cancer biology and the development of new therapeutic strategies.

**Figure 4:**
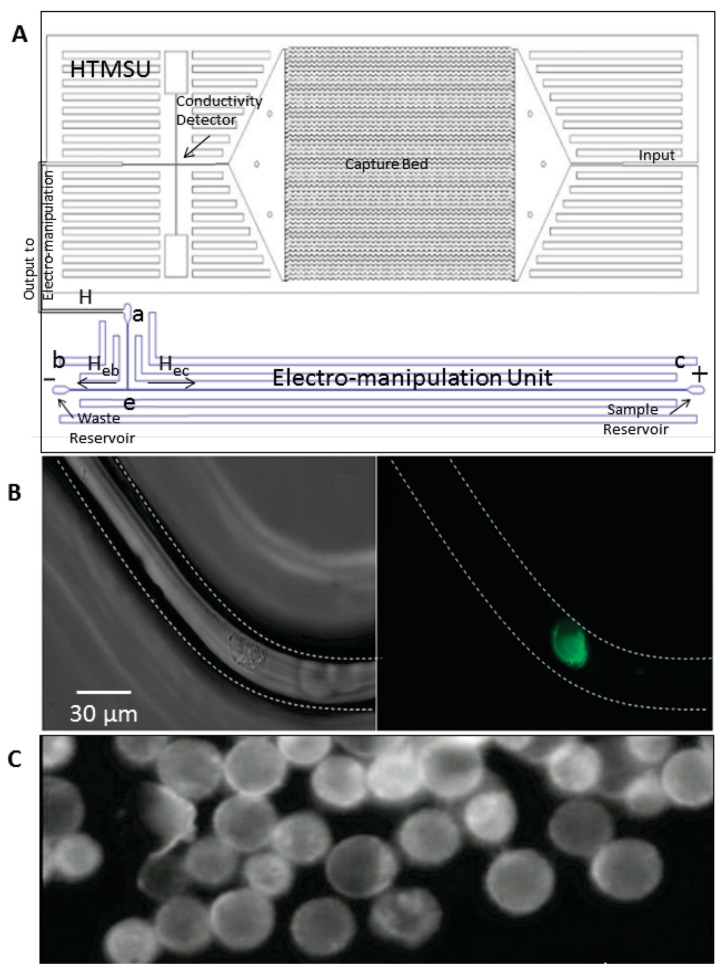
Diagrams showing the microfluidic system, which included the high-throughput microsampling unit and the electromanipulation unit. (**A**) The capture bed consisted of a series of 51 curvilinear channels (30 μm wide and 150 μm deep). The electromanipulation unit contained 80 μm wide, 100 μm deep, and 5 cm long linear channels. Conductometrically enumerated CTCs were introduced into the electromanipulation unit at port a, which served as the entrance port. Port a was connected to a “T” junction labeled e. Exit b served as the sample waste reservoir, while reservoir c was the CTC-receiving reservoir (anodic reservoir). (**B**) Brightfield (**left**) and fluorescence (**right**) micrographs (40×) of selected SW620 CTCs using the high-throughput microsampling unit. (**C**) The selected SW620 CTCs were enriched into reservoir c due to their intrinsic electrophoretic mobility and the applied electric field. The total volume of the receiving reservoir was 2 μL (Copyright © 2011 American Chemical Society. Reprinted from [68] with permission).

One of the primary advantages of electrokinetic microfluidic chips is the precision and control they offer over the cellular microenvironment. These devices can manipulate chemical gradients, fluid dynamics, and cellular interactions with high accuracy, which is crucial for studying the intricate processes involved in cancer progression and metastasis. For example, researchers can use electrokinetic forces to position cancer cells in specific locations within the microfluidic device, creating well-defined microenvironments that closely mimic the in vivo conditions of human tissues [65,66]. This level of control allows for detailed investigations into how cancer cells respond to different stimuli, including drugs, signaling molecules, and mechanical forces. On the other hand, the high-throughput capability of electrokinetic microfluidic chips is another significant advantage [67,68]. These platforms enable the analysis of numerous variables simultaneously, facilitating rapid screenings and extensive data collection. An example of a microfluidic system including high-throughput microsampling unit and the electromanipulation unit is shown in Figure 4. Circulating tumor cells (CTCs) were introduced into the microfluidic system and enriched due to their intrinsic electrophoretic mobility. This is particularly important in cancer research, where understanding the complex interactions between cancer cells and their microenvironment requires the examination of multiple factors. For instance, researchers can use electrokinetic microfluidic chips to conduct high-throughput drug screenings, testing the effects of various compounds on cancer cell viability, proliferation, and migration [69,70]. This approach accelerates the identification of potential therapeutic agents and helps prioritize candidates for further development. Additionally, the integration of imaging technologies within electrokinetic microfluidic systems allows for real-time monitoring of cellular behaviors and responses. High-resolution imaging techniques, such as fluorescence microscopy and live-cell imaging, enable researchers to observe changes in cell morphology, gene expression, and protein localization in response to experimental treatments [71,72,73,74,75]. This real-time observation capability provides immediate insights into the mechanisms of action of different therapies and facilitates the rapid optimization of treatment strategies. For example, by using live-cell imaging, researchers can track the movement of cancer cells within the microfluidic device and study how they interact with surrounding cells and the extracellular matrix [76,77]. This information is invaluable for understanding the dynamic processes involved in cancer invasion and metastasis.

Despite these advantages, electrokinetic microfluidic chips also face several challenges. One of the main disadvantages is the complexity of design and operation. The development and operation of these devices can be complex and require specialized knowledge, which might limit their use to specialized laboratories with the necessary expertise and equipment. Additionally, the integration of advanced imaging and analytical technologies can further increase the complexity and cost of these systems. Scalability is another significant challenge for electrokinetic microfluidic chips. While these devices are excellent for detailed studies and small-scale experiments, scaling them for broader clinical applications or high-throughput industrial uses can be challenging. The precise control and small volumes that are advantageous in research settings can become limitations when attempting to scale up for large-scale drug screenings or clinical diagnostics. Developing methods to produce and operate multiple electrokinetic microfluidic devices simultaneously will be crucial for integrating this technology into high-throughput workflows.

Looking to the future, several directions hold promise for advancing the application of electrokinetic microfluidic chips in cancer research. One potential area of development is the integration of these devices with machine learning algorithms. Machine learning can enhance the analysis of complex data sets generated by microfluidic experiments, improving the predictive accuracy of cellular behavior in response to treatments. Another exciting prospect is the expansion of electrokinetic microfluidic chips to incorporate 3D cell culture systems. While traditional 2D cultures have been valuable for studying cancer biology, they often fail to capture the complexity of the in vivo tumor microenvironment. Three-dimensional cell cultures, on the other hand, provide a more realistic model of tissue architecture and cellular interactions. By advancing beyond 2D cultures, electrokinetic microfluidic devices could increasingly incorporate 3D cell culture systems, offering more relevant insights into cancer biology and treatment responses.

## 6. Microfluidic Chip for the Study of Immune Response

**Figure 5:**
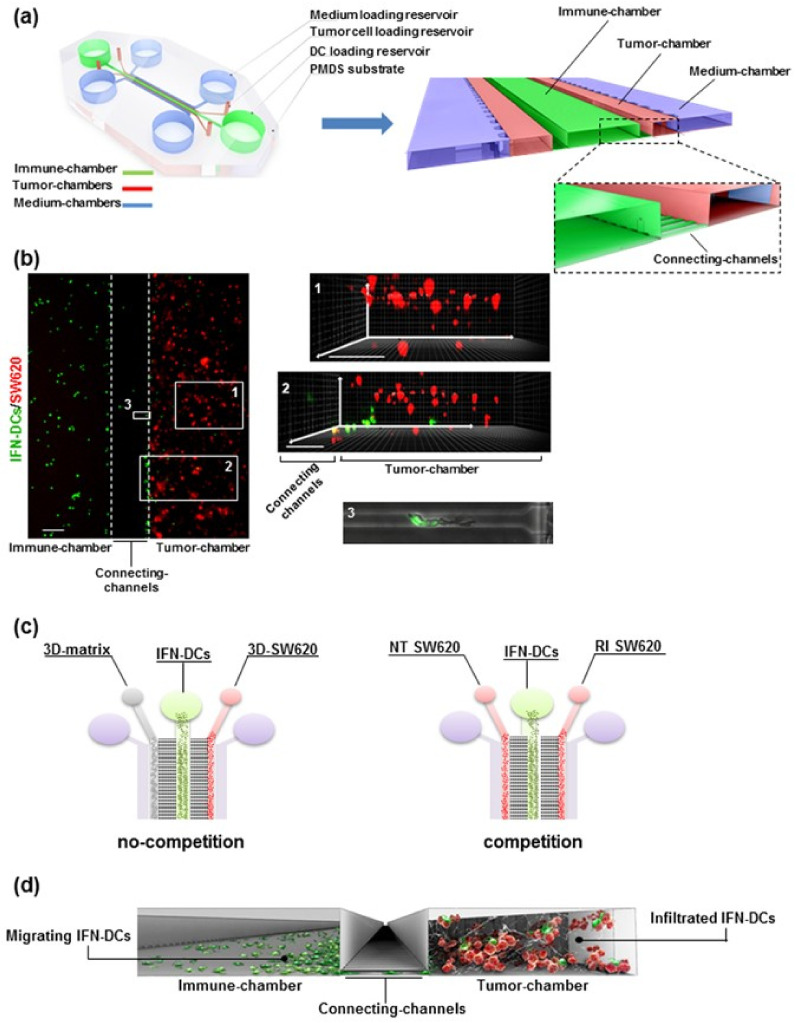
Design of the microfluidic device reconstructing the 3D immune-cancer spaces to track dendritic cells (DC)-cancer cells dialogue. (**a**) Schematic model of the 3D microfluidic device used for real-time monitoring of DC migration toward cancer cells and their interactions. (**b**) Fluorescence image of the immune-tumor area of the device analyzed in microfluidic experiments (left panel) showing the immune chamber filled with PKH67 green-stained DCs, the connecting channels, and the tumor chamber loaded with type I collagen-embedded PKH26 red-stained cancer cells. (**c**) Schematic representation of isometric views of microfluidic devices showing the two experimental configurations used: no-competition and competition settings. (**d**) A 3D graphic representation of a section of the microfluidic device showing the interconnection between immune and tumor spaces and reproducing the motion of migrating DCs from the immune chamber, crossing connecting channels, toward the tumor chamber, where cancer cells are targeted by infiltrated DCs (Copyright © 2017 Parlato et al. Reprinted from [82]).

Microfluidic devices offer unparalleled advantages for cancer research, particularly in unraveling the complexities of the immune response within the tumor microenvironment. These devices allow researchers to replicate the tumor microenvironment with high fidelity, enabling a deeper understanding of the dynamic interplay between cancer cells, immune cells, and other components. By mimicking conditions such as nutrient gradients, mechanical forces, and cellular heterogeneity, microfluidic platforms enable precise studies of how immune cells, such as T cells, macrophages, and dendritic cells, interact with tumor cells. This insight is critical for developing immunotherapies and understanding mechanisms of immune evasion [78,79,80,81,82,83]. For instance, the design of the microfluidic device reconstructing the 3D immune-cancer spaces to track dendritic cells and cancer cells dialogue is shown in Figure 5. The platform replicates tightly interconnected cancer and immune systems within specific 3D environmental conditions to study human DC behavior toward tumor cells. By integrating the microfluidic platform with advanced microscopy and an enhanced cell tracking analysis algorithm, the efficient motion of IFN-DCs toward drug-treated cancer cells was evaluated, as well as subsequent phagocytosis events. Researchers can manipulate parameters such as nutrient gradients, oxygen levels, and mechanical forces with high accuracy. This capability is particularly valuable in studying how cancer cells adapt to hypoxia and develop resistance to treatments. By providing a controlled environment, microfluidic devices facilitate the study of cancer cell behavior under conditions that closely mimic those in the human body. Microfluidic chips also enable real-time observation of cellular interactions, providing immediate insights into the mechanisms of immune responses and therapy effectiveness. High-resolution imaging technologies, such as fluorescence microscopy and live-cell imaging, can be integrated with microfluidic platforms for studying the dynamic processes involved in cancer progression. Microfluidic devices were developed to observe interactions between cancer cells and immune cells in real time, offering new perspectives on how tumors evade immune surveillance and guiding the development of more effective immunotherapies [84,85,86].

Despite these advantages, microfluidic technologies face challenges related to complexity, cost, and scalability. Fabricating intricate microfluidic chips requires advanced techniques and materials, which can drive up costs and limit accessibility. Additionally, the specialized expertise needed to operate and interpret data from these devices can be a barrier to widespread adoption in non-specialized labs. Addressing these issues through the development of user-friendly designs, cost-effective manufacturing methods (e.g., 3D printing or soft lithography), and standardized protocols will be essential to making microfluidic platforms more broadly applicable in cancer research. Expanding microfluidic platforms to incorporate 3D models of tumors is another promising direction. While current systems predominantly rely on 2D structures, 3D microfluidic models provide a more realistic representation of the tumor microenvironment, including spatial architecture and cell-to-cell interactions. These advanced models could improve the predictive accuracy of preclinical studies and reduce the reliance on animal models. Moreover, personalized medicine represents another exciting frontier. Microfluidic devices could be used to test individual patients’ cancer cells against a panel of therapies in real time, enabling the selection of the most effective treatment with minimal side effects. Such applications align with the broader trend toward tailoring treatments to individual patient profiles, potentially improving outcomes and reducing healthcare costs. By addressing current limitations and leveraging emerging opportunities, microfluidic devices have the potential to revolutionize cancer research and therapy development. They provide an unprecedented platform for studying cancer biology, testing innovative therapies, and advancing our understanding of immune–tumor interactions, ultimately contributing to improved patient outcomes and accelerated progress in the fight against cancer.

## 7. Concluding Remarks

Microfluidic technology offers significant advantages for cancer research. This review article summarizes five main areas, including droplet-based microfluidics, organ-on-chip systems, paper-based microfluidics, electrokinetic chips, and immune response analysis. These technologies have their advantages and limitations, as listed in Table 1. Each technology excels in specific domains but has limitations in others, making their use case-dependent. For instance, droplet-based microfluidics and organ-on-chip systems are ideal for cutting-edge biomedical research, while paper-based microfluidics excels in low-resource diagnostics. Electrokinetic chips are suitable for molecular manipulation and detection, whereas microfluidic chips for immune response analysis are particularly valuable in vaccine development and immunotherapy research. These microfluidic devices and systems provide high-fidelity simulations of the tumor microenvironment, dynamic testing capabilities, and real-time observations of cellular processes. Despite challenges related to complexity, cost, and scalability, ongoing advancements in microfluidic technology promise to enhance the precision, scalability, and accessibility of these platforms. Future developments in integrating microfluidic devices with advanced imaging technologies and AI, expanding to 3D models, and applying personalized medicine approaches will further expand the utility of this technology in cancer research. By addressing these challenges and leveraging new opportunities, microfluidic technology has the potential to revolutionize cancer research and therapy development, ultimately improving patient outcomes and advancing our understanding of cancer biology.

## Figures and Tables

**Table 1:** Comparison of the microfluidic technologies in advancing cancer research.

Technologies	Key Applications	Advantages	Limitations
Droplet-based microfluidics	Single-cell analysis, drug discovery	◆ High throughput and automation. ◆ Precise control over volumes. ◆ Compatibility with biochemical and biological samples.	◆ Complex fabrication processes. ◆ Challenges with scalability and droplet stability.
Organ-on-chip systems	Drug testing, disease modeling	◆ Realistic simulation of organ function and disease states. ◆ Reduction of the need for animal testing. ◆ Enablement of long-term studies with dynamic flow conditions.	◆ High complexity and cost of design and operation. ◆ Limited scalability and standardization.
Paper-based microfluidics	Point-of-care diagnostics, 3D tumor modeling	◆ Low-cost, lightweight, and portable. ◆ Easy fabrication using wax printing or cutting. ◆ Minimal reagents and infrastructure required.	◆ Limited sensitivity and resolution compared to other microfluidic platforms. ◆ Restricted to qualitative or semi-quantitative analysis.
Electrokinetic chips	DNA sequencing, biosensing	◆ Precise control over particle movement. ◆ Compatible with integration into lab-on-a-chip systems. ◆ High sensitivity for certain applications.	◆ Specific electrical properties of samples required. ◆ High voltage or heating required that may limit biological sample compatibility.
Microfluidic chips for the study of immune response	Vaccine development, immunotherapy	◆ Enablement of detailed profiling of immune cell populations and cytokine responses. ◆ Advancement in single-cell analysis and multiplexing. ◆ High relevance for understanding disease mechanisms.	◆ High complexity and cost for high-throughput immune profiling. ◆ Specialized equipment and expertise required.
